# Exposomics: perfection not required

**DOI:** 10.1093/exposome/osae006

**Published:** 2024-10-04

**Authors:** Gary W Miller

**Affiliations:** Editor-in-Chief, Exposome, Department of Environmental Health Sciences, Mailman School of Public Health, Columbia University, New York, NY, USA

The enthusiasm for exposomics continues to grow. As with any emerging field, there are challenges to overcome, differences to discuss, and expectations to manage. However, I continue to hear negative comments about the feasibility of exposomics even from those who are supporters. Statements like, “we can’t do the full exposome,” “we will never be able to measure all the components of the exposome,” and “maybe we can do a partial exposome” are misguided and distracting. Let’s start with the definition of exposomics:

Exposomics is the field that studies the comprehensive and cumulative effects of physical, chemical, biological, and psychosocial influences that impact biological systems by integrating data from a variety of interdisciplinary methodologies and streams to enable discovery-based analysis of environmental influences on health.^1^

I challenge anybody to tell me that the above definition is not feasible or attainable. Exposomics is studying the comprehensive and cumulative effects of the range of influences. We are trying to learn as much as possible about these influences, how they interact, where the factors converge, and how they impact our health. Since when has perfection been a requisite in science? A goal perhaps, but compulsory?

Michealangelo’s David is considered one of the greatest works of art in the world. It has been studied by budding artists and even scientists. Detailed anatomical analysis of the sculpture revealed a key back muscle was missing along with several other inaccuracies.^2^ Michealangelo was apparently fully aware of the muscular omission. The piece of marble from which he was working simply did not allow him to recreate it. To criticize Michealangelo’s work because of a few deviations from reality is misplaced. He was not a realist simply trying to imitate nature, he was an artist working within the confines of the resources he had available at the time to create something beautiful.^3^ Had he waited for the perfect piece of marble to be available, he may not have created the David.

I have often stated (in multiple languages) that we should not let perfection be the enemy of the good when it comes to the exposome and exposomics. From a semantic, and potentially pedantic perspective, if one insists that the exposome must be the totality of our exposures from conception onward, one can argue that it is indeed impossible. The original Wild definition of the exposome did not include the word totality^4^; he only referred to this concept in a follow-up paper when discussing potential research strategies.^5^**We should not strive for perfection and we don’t need totality**. We need a counterweight to the genetic drivers of health and disease.^6^ We are attempting to understand the human condition, and without a similarly robust assessment of the non-genetic constituents, our understanding of human biology is limited. Ideally, exposomics develops a framework that allows as much data to be incorporated as possible. For some populations, it may be limited to years of exposure; for others, it could be a lifetime. Our exposomes are living documents that continue to evolve. Yet, at any given point in time, decisions must be made about prevention, treatment, or policy and those decisions must be made with the information available at that point in time. Waiting for the mythical everything or totality will leave us in a state of perpetual indecision.

The Human Genome Project was a Herculean undertaking that was not without controversy.^7-14^ We celebrated the sequencing of first human genome (nearly complete) with a ceremony at the White House ceremony full of dignitaries in 2000 (Tony Blair, William Clinton, Francis Collins, Craig Venter, Ruth Kirschstein, James Watson, Aristides Patrinos). The Human Genome Project was then claimed to be “officially” completed in 2003 and reported in companion issues of Science and Nature.^15^^,^^16^ However, it wasn’t until the completion of the Telomere-to-Telomere Consortium in 2022 that Eric Green, Director of NHGRI, declared that the human genome was truly complete.^17^^,^^18^ Even though the human genome wasn’t complete until 2022, that did not prevent the field from making major advances in 2005, 2010, or 2015. The incomplete knowledge of the human genome was actionable, truly awesome, and could be considered a scientific masterpiece akin to Michaelangelo’s David. For decades, it was not perfect, and even today, we have not achieved totality given that mutations and modifications to our genomes occur on a daily basis. We are still learning about our genomes and their inherent variations, as we are still learning about the universe in which we live and that is what science is—the relentless pursuit of knowledge, the accumulation of information, and the assimilation of the findings into coherent intellectual models.

The emerging definition of the exposome is the integrated compilation of all physical, chemical, biological, and psychosocial influences that impact biology.^1^ Can we ever figure out *all* of the influences? Probably not, but is it necessary? If we focus our efforts on greatly expanding our understanding of the exposures we face throughout our lives, we will improve our understanding of human health and disease, which will enhance our ability to make data-driven decisions. Providing a comprehensive and systematic analysis of the environmental influences on health will be a masterpiece as beautiful as what genetics and genomics have accomplished.

It is simply counterproductive to state that we can never measure the human exposome or we can’t effectively use exposomics if we can’t do everything. We should be self-critical, we should pursue the most rigorous and harmonized science, and we should strive to be as comprehensive and systematic as possible. But leave your desire for perfection at the door, and strive to create a work of scientific art.
